# Soybean *CALCIUM-DEPENDENT PROTEIN KINASE17* Positively Regulates Plant Resistance to Common Cutworm (*Spodoptera litura* Fabricius)

**DOI:** 10.3390/ijms232415696

**Published:** 2022-12-10

**Authors:** Huiqi Wang, Xiao Li, Fenglin Su, Hailun Liu, Dezhou Hu, Fang Huang, Deyue Yu, Hui Wang

**Affiliations:** 1 National Center for Soybean Improvement, National Key Laboratory of Crop Genetics and Germplasm Enhancement, Jiangsu Collaborative Innovation Center for Modern Crop Production, Nanjing Agricultural University, Nanjing 210095, China; 2 Key Laboratory of South Subtropical Fruit Biology and Genetic Resource Utilization, Guangdong Provincial Key Laboratory of Tropical and Subtropical Fruit Tree Research, Institute of Fruit Tree Research, Guangdong Academy of Agricultural Sciences, Ministry of Agriculture and Rural Affairs, Guangzhou 510640, China

**Keywords:** soybean, *GmCDPK17*, resistance, common cutworm, genetic diversity

## Abstract

Soybean is frequently attacked by herbivorous pests throughout the growth period. Exploring anti-insect genes to improve insect resistance in soybean is an important soybean breeding goal. Here, we cloned and characterized the gene for a quantitative trait locus (QTL) related to insect resistance, *Glyma.06g189600*, which encodes CALCIUM-DEPENDENT PROTEIN KINASE17 (*GmCDPK17*) in soybean. The pairwise sequence alignment analysis revealed that the presumed protein of *GmCDPK17* shares 52.06% similarity with that of *GmCDPK38*, a known negative regulatory gene of insect resistance in soybean. Ectopic expression of *GmCDPK17* and *GmCDPK38* restored the phenotypes of the Arabidopsis insect-susceptible mutant *cpk10* and insect-resistant mutant *cpk28*, respectively. Moreover, transgenic hairy roots of the soybean cultivar Jack were generated by *Agrobacterium*-mediated transformation. Overexpression of *GmCDPK17* increased soybean hairy root resistance to common cutworm (CCW), while RNA interference of the gene decreased soybean hairy root resistance to CCW. Sequencing data from the cultivated and wild soybeans were used to analyze the genetic diversity of *GmCDPK17*. This gene was subjected to domestication selection. Six and seven haplotypes (Haps) were identified in cultivated and wild soybeans, respectively. The resistance Hap1 is not widely used in cultivated soybeans and is mainly distributed at low latitudes. Accessions with resistance haplotypes of the *GmCDPK17* and *GmCDPK38* genes showed high resistance to CCW. Altogether, we revealed a novel positive regulatory insect resistance gene, *GmCDPK17*, which may further improve insect resistance in soybean.

## 1. Introduction

As an important crop, soybean (*Glycine max*) provides high-quality oil and protein for humans and other animals. However, soybean plants are often attacked by insects throughout the growth period. The common cutworm (CCW) (*Spodoptera litura* Fabricius) is one of the major herbivorous insects that attack soybean plants in southern China [1]. CCW may reduce soybean seed yields by as much as 50–100% without any control measures [2]. Therefore, improving soybean insect resistance by exploring and adapting endogenous insect resistance genes is an important soybean breeding goal and an effective insect management practice in soybean.

Several endogenous insect resistance genes in soybean have been reported. Most of them are obtained using a homologous cloning strategy, such as the allene oxide synthase (AOS) gene *GmAOS* [3], allene oxide cyclase (AOC) gene *GmAOC3* [4], MYC2-like basic helix-loop-helix Leu zipper transcription factor *GmMYC1* [5], and terpene synthase (TPS) gene *GmTPS3* [6]. The transgenic tobacco strains expressing these genes all acquire greater resistance to CCW than nontransgenic controls. Overexpression of *GmAOS* and *GmAOC3* in tobacco increases the synthesis and level of jasmonic acid-related compounds [3,4]. *MYC2* positively regulates the expression of jasmonic acid-responsive insect resistance genes in Arabidopsis [7]. TPSs catalyze the formation of monoterpenes and sesquiterpenes [8,9], which are key plant defense components. With the development of soybean genome research, a few insect resistance genes have been obtained from quantitative trait loci (QTLs) in recent years. For example, *G*m*UGT* negatively regulates soybean resistance to CCW [10]. The gene encodes a UDP-glycosyltransferase (UGT) and is the marker gene for QTL-M in soybean.

In plants, calcium signaling is involved in defending against pests [11]. Upon sensing touch- and wounding-related signals, the ion channels on the plasma membrane of plant cells are first activated, and the concentration of calcium (Ca^2+^) ions in the cytoplasm changes rapidly, activating additional signal transduction pathways and inducing the expression of defense- or growth-related genes [12,13,14]. Calcium-dependent protein kinase (CDPK) is one of the Ca^2+^ sensors in plants that transduces Ca^2+^ signals by phosphorylating specific substrates [15,16]. The structure of CDPKs consists of a typical Ser/Thr protein kinase domain, a calmodulin (CaM)-like domain (including EF-hand Ca^2+^-binding sites), a variable N-terminal domain, and a junction domain with autoinhibitory properties [17].

The plant *CDPK* gene family is large. Notably, 50, 35, 34, 31, and 26 *CDPK* genes have been identified in soybean, maize (*Zea mays*), Arabidopsis, rice (*Oryza sativa*), and wheat (*Triticum aestivum*), respectively [18,19,20,21]. The 50 *CDPKs* of soybean were grouped into four major subfamilies (I-IV) based on a phylogenetic analysis, as those of other plants [18,20,22]. In subfamily IV, *GmCDPK38* gene has been shown to be associated with insect resistance. Knockout of *GmCDPK38* increased soybean resistance to CCW [23]. The homologous genes of *GmCDPK38* in Arabidopsis and native tobacco (*Nicotiana attenuata*) are *CPK28* and *NaCDPK4/5*, respectively [24]. The *cpk28* mutant exhibited growth reduction and ectopic lignifications in stems and enhanced resistance against bacterial infection [25,26]. Simultaneous silencing of *NaCDPK4* and *NaCDPK5* resulted in a massive accumulation of jasmonic acid and increased resistance to insects [27]. In subfamily III, the Arabidopsis *cpk10* mutant exhibited reduced expression of the defense related genes *pathogenesis-related 1* (*PR1*), *PR2* and *avrRpt2 induced gene 1* (*AIG1*) after inoculation with pathogens [28], while *CPK3* and *CPK13* activate the wound- and herbivore-induced network by the accumulation of *plant defensin* (*PDF1.2*) [29]. However, direct evidence for the roles of these subfamily III members in plant insect resistance is still lacking. In addition, few studies have focused on comparing the roles of different *CDPK* subfamily genes in insect resistance.

In our previous research, we performed a genome-wide association analysis to map a QTL associated with soybean resistance to CCW on chromosome 6 [30]. In the present study, we cloned the *GmCDPK17* gene from the QTL. By analyzing the expression patterns, function, and genetic diversity of the gene and comparing them with those of *GmCDPK38*, we discovered a new gene, *GmCDPK17*, that positively regulated soybean resistance to insects, was selected by domestication, was not widely used in cultivated soybeans, and was different from *GmCDPK38* in soybean resistance to CCW. The discovery of the gene is helpful to further enhance insect resistance in soybean.

## 2. Results

### 2.1. Identification of Candidate Genes

**Figure 1:**
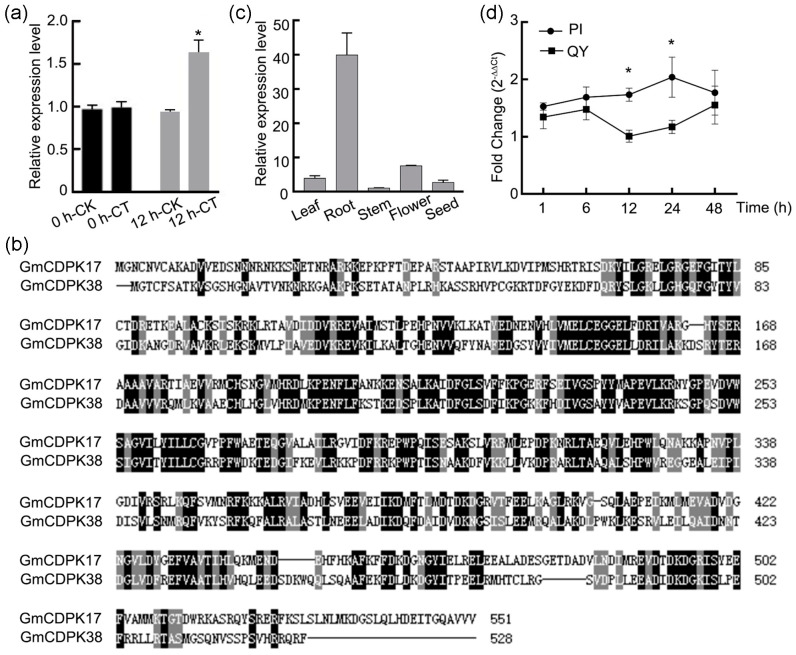
Induced expression, tissue expression, and sequence analysis of *GmCDPK17* (*Glyma.06g189600*). (**a**) CCW-induced expression of *GmCDPK17* at 0 and 12 h. 0 h-CK/CT, control and treatment before CCW induction. 12 h-CK/CT, control and treatment after CCW induction for 12 h (*n* = 3). (**b**) Amino acid sequence alignment of *GmCDPK17* and *GmCDPK38*. (**c**) Analysis of *GmCDPK17* expression in different soybean tissues (*n* = 3). The relative expression levels are normalized to that of the *GmTubulin* gene and relative to the expression in the stem (relative expression level in the stem = 1). (**d**) CCW-induced expression of *GmCDPK17* at different time points. The fold change of *GmCDPK17* transcript levels at 1, 6, 12, 24, and 48 h after CCW attack were calculated by comparing gene expression levels under induction and noninduction at the same timepoint (*n* = 3). The relative expression levels are normalized to that of the *GmTubulin* gene and relative to the expression in control plants at 1 h (relative expression level in control plants at 1 h = 1). Error bars denote ±SE. Two-tailed *t*-test was used for all statistical analyses: * *p* < 0.05. All error bars denote ±SE.

Three single-nucleotide polymorphisms (SNPs) significantly associated with larval weight and larval duration were detected on chromosome 6 [30]. These SNPs are located in one locus containing 18 genes (Supplementary Table S1). Of them, six genes were found to participate in the stress response and signal transduction, including *Glyma.06g189500*, *Glyma.06g189600*, *Glyma.06g190200* and *Glyma.06g190800* (Supplementary Table S1). Ten genes of the 18 genes were expressed at high levels in distinct tissues of soybean, namely, *Glyma.06g189500*, *Glyma.06g189600*, *Glyma.06g189700*, *Glyma.06g189900*, *Glyma.06g190000*, *Glyma.06g190200*, *Glyma.06g190300*, *Glyma.06g190900*, *Glyma.06g191000* and *Glyma.06g190200* (Supplementary Table S1). Then, a quantitative real-time polymerase chain reaction (qRT-PCR) analysis was performed to determine the expression patterns of these 18 genes in soybean leaves after CCW attack. As shown in Figure 1a and Figure S1, only the *Glyma.06g189600* gene was significantly upregulated. Based on these results, *Glyma.06g189600* was used as a candidate gene of the locus, and its function was further studied. The gene encodes a calcium-dependent protein kinase (Supplementary Table S1) belonging to subgroup III of soybean CDPKs and was named *GmCDPK17* according to Hettenhausen et al. [18].

### 2.2. Cloning and Sequence Analysis of GmCDPK17

The full sequence of the *GmCDPK17* CDS is 1656 bp and encodes 551 amino acids with an estimated molecular mass of 62.5 kDa (Figure S2a). The predicted amino acid sequence of the gene contains a serine/threonine protein kinase catalytic domain and four EF chiral calcium-binding motifs, consistent with the typical structural characteristics of the calcium-binding protein kinase family (Figure S2b). The pairwise sequence alignment analysis revealed that GmCDPK17 shares moderate amino acid sequence similarity with GmCDPK38 (52.06%) (Figure 1b).

### 2.3. Expression Patterns of GmCDPK17 in Distinct Tissues and Leaves after CCW Attack

The expression patterns of *GmCDPK17* were identified in different soybean tissues and leaves at multiple time points after CCW induction using qRT-PCR analysis. The *GmCDPK17* gene was expressed at high levels in roots, flowers, and leaves (Figure 1c). Moreover, the *GmCDPK17* gene was constantly upregulated after CCW induction and peaked at 24 h in the resistant material PI 227687 (PI) (Figure 1d). However, in the susceptible material Qinyangdadou (QY), the expression of the *GmCDPK17* gene was initially upregulated, decreased at 12 h, and then was upregulated again. In addition, the expression levels of the gene in QY were lower than those in PI. These results suggested that the *GmCDPK17* gene may be involved in the defense response of soybean to CCW.

### 2.4. GmCDPK17 Localized to the Nucleus and Cytoplasm

**Figure 2:**
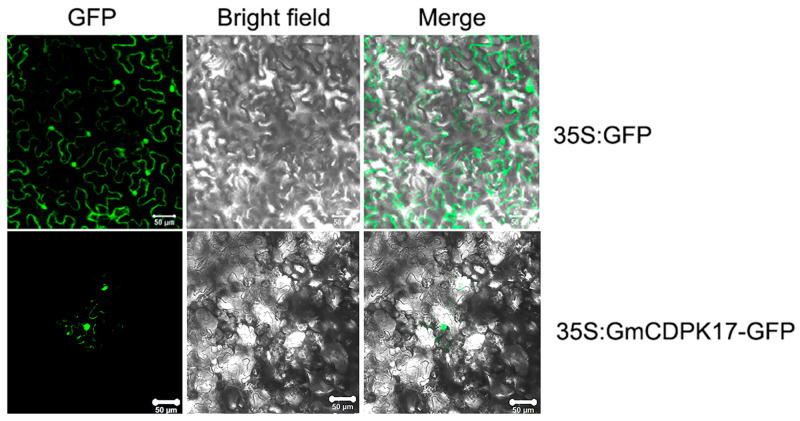
Subcellular localization of GmCDPK17 in tobacco mesophyll cells. GFP: green fluorescence protein; Scale bars: 50 μm.

The CaMV 35S promoter was used to express the fusion protein encoded by the GmCDPK17 CDS and the green fluorescent protein (GFP) sequence to map the location of GmCDPK17 in cells. The recombinant vector 35S:GmCDPK17-GFP was transformed into tobacco mesophyll cells instantaneously. Subcellular localization results showed that the fusion protein was localized to the nucleus and cytoplasm, and the GFP signal was detected throughout the control cells transformed with the empty vector 35S:GFP (Figure 2).

### 2.5. The Roles of GmCDPK17 in Arabidopsis Development and Resistance to CCW Differed from GmCDPK38

**Figure 3:**
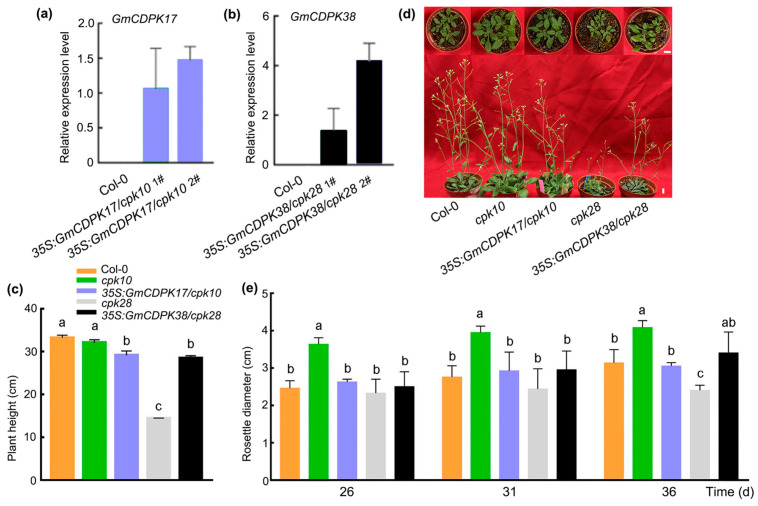
Phenotypes of Arabidopsis Col-0, *cpk10*, *35:GmCDPK17/cpk10*, *cpk28*, and *35S:GmCDPK38/cpk28*. (**a**) qRT-PCR analysis of *GmCDPK17* expression in Col-0 and two *35S:GmCDPK17/cpk10* plants at 30 d after being transferred from MS medium to soil (*n* = 3). The relative expression levels were normalized to that of the *AtTubulin* gene and are reported relative to the expression in transgenic Arabidopsis line 1 (*35S:GmCDPK17/cpk10* #1) (relative expression level in *35S:GmCDPK17/cpk10* #1 = 1). (**b**) qRT-PCR analysis of *GmCDPK38* expression in Col-0 and two *35S:GmCDPK38/cpk28* plants (*n* = 3). The relative expression levels were normalized to that of the *AtTubulin* gene and are reported relative to the expression in transgenic Arabidopsis line 1 (*35S:GmCDPK38/cpk28* 1#) (relative expression level in *35S:GmCDPK38/cpk28* 1# = 1). (**c**) Plant height of 36-d-old Arabidopsis seedlings. (**d**) Twenty-one-d-old (upper panel) and 31-d-old (lower panel) Arabidopsis seedlings. Scale bars: 1 cm. (**e**) Rosette leaf diameter of 26-d-old, 31-d-old, and 36-d-old Arabidopsis seedlings. Lowercase letters above the bars in the histograms denote intragroup significance at *p* < 0.05. The different lowercase letters and same lowercase letters represent significant differences and nonsignificant differences, respectively. Two-tailed *t*-test was used to generate the *p* values. All error bars denote ± SE.

*GmCDPK17* and *GmCDPK38* were ectopically expressed in the Arabidopsis *cpk10* and *cpk28* mutants, respectively, to compare the effects of *GmCDPK17* and *GmCDPK38* on plant resistance to insects. *GmCDPK17* and *GmCDPK38* transgenic lines, *35S:GmCDPK17/cpk10* and *35S:GmCDPK38/cpk28*, with relatively high expression were selected for further phenotypic analysis (Figure 3a,b and Figure S3). The Arabidopsis ecotype Col-0 was used as a control. Compared with Col-0, the mutant *cpk10* line had a longer rosette diameter and similar stem height, but the mutant *cpk28* line showed a shorter rosette diameter and stem height (Figure 3c–e). The growth reduction phenotype of the *cpk28* mutant is consistent with the previous report [25]. *35S:GmCDPK17/cpk10* T_1_ and *35S:GmCDPK38/cpk28* T_1_ were not significantly different from Col-0 in rosette diameter but showed a shorter stem height than Col-0 (Figure 3c–e).

**Figure 4:**
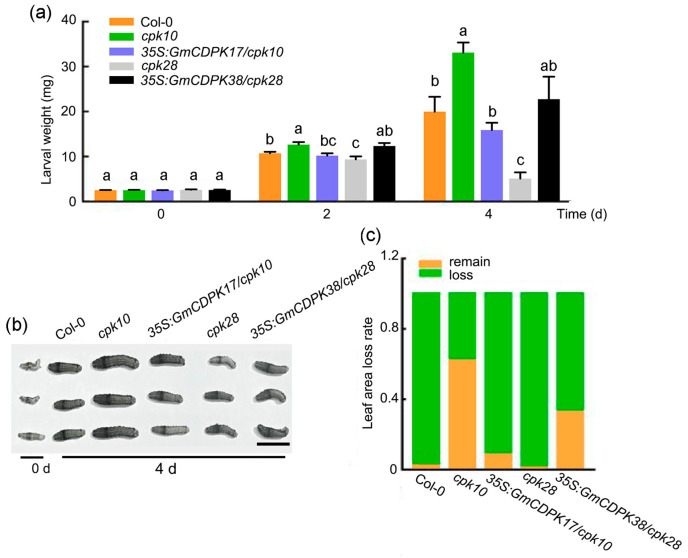
Identification of the Arabidopsis Col-0, *cpk10*, *35:GmCDPK17/cpk10*, *cpk28*, and *35S:GmCDPK38/cpk28* resistance to CCW. (**a**) The weight of larvae fed Col-0, *cpk10*, *35S:GmCDPK17/cpk10*, *cpk28* and *35S:GmCDPK38/cpk28* at 0, 2 and 4 d (*n* = 3). Lowercase letters above the bars in the histograms denote intragroup significance at *p* < 0.05. The different lowercase letters and same lowercase letters represent significant differences and nonsignificant differences, respectively. Two-tailed *t*-test was used to generate the *p* values. The error bar represents the ±SE. (**b**) CCW larvae fed Col-0, *cpk10*, *35S:GmCDPK17/cpk10*, *cpk28* and *35S:GmCDPK38/cpk28* at 0 and 4 d. Scale bar: 1 cm. (**c**) Leaf area loss rate at 12 h after CCW feeding.

Force-feeding tests and free-feeding tests were conducted to assess the insect resistance of Col-0, *cpk10*, *cpk28*, *35S:GmCDPK17/cpk10* T_1_, and *35S:GmCDPK38/cpk28* T_1_. Five Arabidopsis plants from each line were used in the two tests. In force-feeding tests, compared with CCW larvae fed on Col-0 plants, those fed on the mutant *cpk10* plants were heavier at 2 and 4 d, whereas those fed on the mutant *cpk28* plants were lighter at 2 and 4 d (Figure 4a,b). No significant difference was observed between the CCW larvae fed on Col-0, the *35S:GmCDPK17/cpk10* T_1_, and *35S:GmCDPK38/cpk28* T_1_ plants (Figure 4a,b). In the free-feeding tests, the leaf area loss rate of *cpk10* was the largest of the five lines and was significantly higher than that of Col-0 after 12 h of feeding (Figure 4c). Col-0, *cpk28*, *35S:GmCDPK17/cpk10*, and *35S:GmCDPK38/cpk28* showed no significant difference in the leaf area loss rate (Figure 4c). Therefore, the mutant *cpk10* was more vulnerable to CCW. Taken together, overexpression of *GmCDPK17* and *GmCDPK38* in *cpk10* and *cpk28*, respectively, restored the phenotype of the mutants.

Based on these results, *GmCDPK17* and *GmCDPK38* played important roles in plant resistance to CCW and in growth and development in Arabidopsis, and the effect of *GmCDPK17* was different from that of *GmCDPK38*.

### 2.6. GmCDPK17 Positively Regulates Soybean Resistance to CCW

**Figure 5:**
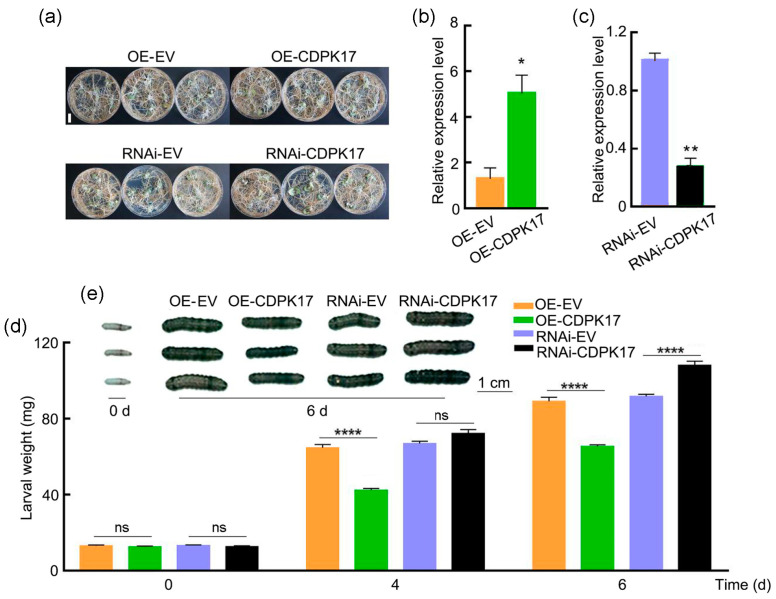
Identification of soybean hairy root resistance to CCW. (**a**) Thirty-day-old soybean hairy roots of the OE-CDPK17, OE-EV, RNAi-CDPK17, and RNAi-EV genotypes. Scale bar: 2 cm. (**b**) Relative expression levels of *GmCDPK17* in OE-CDPK17 and OE-EV transgenic soybean hairy roots. The relative expression level is the expression level of OE-EV (expression level 1) standardized to that of the *GmTubulin* gene (*n* = 3). (**c**) Relative expression levels of *GmCDPK17* in RNAi-CDPK17 and RNAi-EV transgenic soybean hairy roots. The relative expression level is the expression level of RNAi-EV (expression level 1) standardized to that of the *GmTubulin* gene (*n* = 3). (**d**) The weight of larvae fed OE-EV, OE-CDPK17, RNAi-EV and RNAi-CDPK17 hairy roots at 0, 4, and 6 d. (**e**) Larvae fed OE-EV, OE-CDPK17, RNAi-EV and RNAi-CDPK17 hairy roots at 0 and 6 d. Scale bar: 1 cm. Two-tailed *t*-test was used for all statistical analyses: * *p* < 0.05, ** *p* < 0.01, **** *p* < 0.0001, ns: not significant. All error bars denote ± SE.

Recombinant overexpression and RNA interference vectors for the gene and the corresponding empty vectors were transformed into soybean hairy roots to evaluate the function of *GmCDPK17* in soybean resistance to CCW. A total of 33 dishes with soybean transgenic hairy roots were obtained for each vector (Figure 5a). PCR was used to identify positive transgenic soybean hairy roots (Figure S4). The expression level of the *GmCDPK17* gene in hairy roots overexpressing this gene (OE-CDPK17) was significantly higher than that in control roots expressing the empty vector pMDC83 (OE-EV). In contrast, the expression level of *GmCDPK17* in hairy roots subjected to RNA interference (RNAi) was significantly lower than that in control roots transformed with the empty vector pB7GWIWG2 (RNAi-EV) (Figure 5b,c).

The weight of larvae fed on hairy roots of OE-CDPK17 was significantly lower than that of larvae fed on OE-EV hairy roots at 4 and 6 d. The weight of larvae fed on hairy roots of RNAi-CDPK17 was significantly higher than that of larvae fed on hairy roots of RNAi-EV at 6 d (Figure 5d,e). The results suggest that the *GmCDPK17* gene positively regulates the resistance of soybean to CCW.

### 2.7. GmCDPK17 Underwent Selection during Soybean Domestication

**Figure 6:**
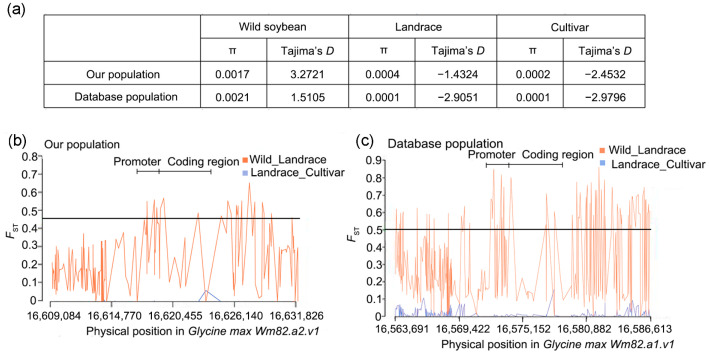
Selection during domestication of the *GmCDPK17* gene. (**a**) *π* value and Tajima’s *D* value of the *GmCDPK17* gene. (**b**) *F*_ST_ values of the *GmCDPK17* gene in our population. (**c**) *F*_ST_ values of the *GmCDPK17* gene in database population. The black line indicates the genome-wide threshold.

We calculated the nucleotide diversity of *GmCDPK17* in our own population and database populations to further investigate whether *GmCDPK17* is a domesticated gene. In the population in our laboratory, wild soybean had the highest *π* value, followed by the local varieties and the cultivated varieties. In 302 database populations, wild soybean also had the highest *π* value, followed by local varieties and cultivated varieties. In addition, Tajima’s *D* value of the *GmCDPK17* gene in both local and cultivated varieties was negative (Figure 6a). The *F*_ST_ values for the coding region of the *GmCDPK17* gene and the upstream and directly downstream regions of the promoter were the lowest in cultivated varieties, followed by local varieties and the highest in wild soybeans (Figure 6b,c). Thus, the *GmCDPK17* gene was selected during soybean domestication.

### 2.8. Genetic Diversity of the GmCDPK17 Gene

**Figure 7:**
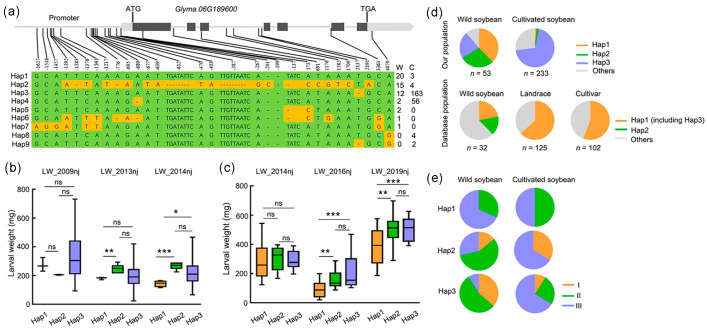
Variation analysis of cultivated soybeans and wild soybeans in our population and domestication selection of *GmCDPK17*. (**a**) Haplotypes of the *GmCDPK17* gene. ‘W’ and ‘C’ represent wild soybean and cultivated soybean, respectively. (**b**) Boxplots of the larval weight of CCW fed cultivated soybeans with haplotypes Hap1, Hap2, and Hap3. LW_2009nj, LW_2013nj, and LW_2014nj represent the evaluated resistance of cultivated soybeans grown in 2009, 2013 and 2014 in Nanjing (nj), respectively, to CCW. (**c**) Boxplots of the larval weight of CCW fed wild soybeans with haplotypes Hap1, Hap2, and Hap3. LW_2014nj, LW_2016nj, and LW_2019nj represent the evaluated resistance of wild soybeans grown in 2014, 2016 and 2019 in Nanjing (nj), respectively, to CCW. (**d**) Distribution of haplotypes Hap1, Hap2 and Hap3 in cultivated and wild soybeans. (**e**) Geographical distribution of haplotypes Hap1, Hap2 and Hap3 in China. I represents northern China, II represents the Huang-Huai-Hai region, and III represents southern China. For all boxplots, the center line is the median; the box limits are the upper and lower quartiles; and the whiskers are the 1.5×interquartile range. Two-tailed *t*-test was used for all statistical analyses: * *p* < 0.05, ** *p* < 0.01, *** *p* < 0.001, ns: not significant.

The approximately 6.9-kb sequence of the *GmCDPK17* gene was analyzed in a sample including 219 cultivated soybeans and 121 wild soybeans. A total of 30 SNPs and insertion-deletions (InDels) (minor allele frequency > 0.05) were identified in the sample (Supplementary Tables S2 and S3). Based on these polymorphic loci, nine haplotypes were identified in cultivated soybean and wild soybean (Figure 7a). Of them, four haplotypes were shared by the two subpopulations. Hap3 and Hap4 were the major haplotypes in cultivated soybean, and Hap1, Hap2, and Hap3 were the major haplotypes in wild soybean (Figure 7a). The larval weight of CCW fed on Hap1 was significantly heavier than that of CCW fed on Hap2 in both environments (LW_2013nj and LW_2014nj) and that of CCW fed on Hap3 in one environment (LW_2014nj) of cultivated soybean. Similarly, the larval weight of CCW fed on Hap1 was significantly different from that of CCW fed on Hap2 and Hap3 in wild soybean in both environments (LW_2016nj and LW_2019nj) (Figure 7b,c). Therefore, Hap1 was the resistance haplotype of *GmCDPK17*, and Hap 2 and Hap3 were the susceptible haplotypes.

Using the data from our 393 resequencing samples to analyze the frequencies of Hap1, Hap2 and Hap3, we found that the frequencies of Hap1 and Hap2 in cultivated soybeans were much lower than those in wild soybeans. In contrast, Hap3 was more frequent in cultivated soybeans than in wild soybeans (Figure 7d). An analysis of the geographical distribution showed that Hap1 was mainly distributed in southern China. Hap2 and Hap3 in wild soybean were distributed mainly in the Huang-Huai-Hai region, and the two haplotypes in cultivated soybeans were distributed mainly in southern China (Figure 7e).

The frequencies of the haplotypes Hap1, Hap2 and Hap3 were also analyzed using sequencing data for 302 soybean varieties from a public database (Supplementary Table S4). Of the 30 SNP and InDel loci identified in our database, 21 were also observed in the database population. Based on these results, Hap2 might be isolated independently, but only in the wild soybean population (5/32); Hap1 and Hap3 were indistinguishable, and the frequency of Hap1 (including Hap3) was relatively low in the wild soybean population (7/32) but high in the local varieties (79/125) and cultivated varieties (57/102) (Figure 7d). These results suggest that *GmCDPK17* lost its resistance haplotype during domestication.

### 2.9. Soybean with Resistance Haplotypes of the GmCDPK17 and GmCDPK38 Genes Showed High Resistance to CCW

**Figure 8:**
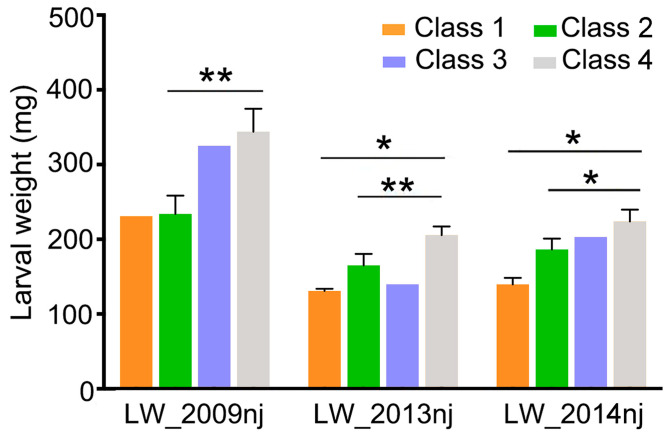
Larval weight of CCW fed cultivated soybean materials with different haplotype combinations. LW_2009nj, LW_2013nj, and LW_2014nj represent the evaluated resistance of cultivated soybeans grown in 2009, 2013 and 2014 in Nanjing (nj), respectively, to CCW. Class I: materials with Hap1 of *GmCDPK17* and Hap2 of *GmCDPK38*, *n* = 1 in LW_2009nj and 2 in LW_2013nj and LW_2014nj; Class II: materials with Hap2 and Hap3 of *GmCDPK17* and Hap2 of *GmCDPK38*, *n* = 13 in LW_2009nj and 14 in LW_2013nj and LW_2014nj; Class III: materials with Hap1 of *GmCDPK17* and Hap3 of *GmCDPK38*, *n* = 1 in LW_2009nj, LW_2013nj and LW_2014nj; Class IV: materials with Hap2 and Hap3 of *GmCDPK17* and Hap3 of *GmCDPK38*, *n* = 14 in LW_2009nj and 19 in LW_2013nj and LW_2014nj. Two-tailed *t*-test was used for all statistical analyses: * *p* < 0.05, ** *p* < 0.01.

The genetic diversity of *GmCDPK38* has been analyzed by Li et al. [23]. Hap2 and Hap3 were the resistance and susceptibility haplotypes of the gene, respectively. In this study, the same soybean populations were used to analyze the haplotypes of *GmCDPK17*, and the resistance haplotype Hap1 and susceptibility haplotypes Hap2 and Hap3 were identified (Figure 7). Based on haplotype combinations of *GmCDPK17* and *GmCDPK38*, 36 cultivated soybeans were identified and grouped into four classes to evaluate the anti-insect effects of different haplotype combinations and reveal the elite genotype combinations of the two genes. Seven cultivated soybeans without insect resistance data were not used for the following analysis in 2019. Class I included accessions (1 in 2009 and 2 in 2013 and 2014) with resistance haplotypes of the two genes, Hap1 of *GmCDPK17* and Hap2 of *GmCDPK38*. Class II included accessions (13 in 2009 and 14 in 2013 and 2014) with Hap2 or Hap3 (susceptibility haplotypes) of *GmCDPK17* and Hap2 (resistance haplotype) of *GmCDPK38*. Class III included accessions (1 in 2009, 2013 and 2014) with Hap1 (resistance haplotype) of *GmCDPK17* and Hap3 (susceptibility haplotype) of *GmCDPK38*. Class IV included accessions (14 in 2009 and 19 in 2013 and 2014) with susceptible haplotypes of two genes, Hap2 or Hap3 of *GmCDPK17* and Hap3 of *GmCDPK38*. The insect resistance of soybean was compared between different classes. As shown in Figure 8, CCWs fed soybeans from Class I and Class II were significantly lighter than those fed soybeans from Class IV in two or more environments. Although a significant difference in larval weight was not observed between accessions of Class I and Class II and the data from Class III were not used to assess significance, the larval weight of CCW fed soybean in Class I was the lowest, followed by the larval weight of CCW fed soybean in Class II and III, and the larval weight of CCW fed soybean in Class IV was the highest. Based on these results, soybean accessions with resistance haplotypes of the two genes were the most insect resistant, followed by those with one gene resistance haplotype. Because only Class IV was found in wild soybean, no further analysis was performed using this subpopulation.

## 3. Discussion

### 3.1. GmCDPK17 Has a Different Function from GmCDPK38 in Soybean Insect Resistance

In the present study, the soybean *GmCDPK17* gene was cloned from a QTL related to insect resistance on chromosome 6. This gene belonged to CDPK subgroup III, and it has a different function from *GmCDPK38* of subgroup IV in soybean insect resistance. Soybean knockout mutants of *GmCDPK38* exhibited significantly increased resistance to CCW [23]. Here, we revealed that the *GmCDPK17* gene positively regulated plant resistance to CCW by transforming Arabidopsis mutants and soybean hairy roots (Figure 4 and Figure 5). The subfunctions of the *GmCDPK17* and *GmCDPK38* genes from different subgroups regarding plant insect resistance may be due to the low amino acid sequence similarity of the two genes. Similarly, Valmonte et al. [22] observed high sequence similarity between genes in subgroups I through III, but the sequences were quite different from those of genes in subgroup IV, as shown by an evolutionary analysis of multiple plant CDPKs. In terms of regulation, Bredow et al. [31] discovered that the CDPKs from subgroup IV have a conserved phosphorylation site (Ser318), but this site is not present in the CDPKs from the other three subgroups, indicating that different subgroup members may be regulated by different factors. These results suggest that the insect resistance mechanism of *GmCDPK17* may differ from that of *GmCDPK38* in soybean. For *GmCDPK38*, the relevant work has been carried out. The knockout of this gene in soybean increased the expression level of defense-related genes and altered the phosphorylation level of defense-related proteins of which the S-adenosylmethionine synthase GmSAMS1 involved in CCW resistance in soybean is identified as a potential target of GmCDPK83 [23,32]. Few homologous genes of *GmCDPK17* in CDPK subgroup III have been reported to participate in plant insect resistance, so the information for speculating the mechanism of *GmCDPK17* is limited. Generally, calcium signaling is upstream of the jasmonic acid signaling pathway in plant response to insect attack [12]. As a member of calcium signaling, *GmCDPK17* may be involved in soybean insect resistance through regulating genes in the jasmonic acid signaling pathway. Therefore, more work is required to reveal the resistance mechanism of *GmCDPK17* and its difference from that of *GmCDPK38*.

### 3.2. GmCDPK17 May Further Improve Soybean Insect Resistance via Polymerization with GmCDPK38

Over the past 20 years, the adoption of transgenic breeding technology has significantly increased world agricultural productivity, grain production and farmer profits, especially the cultivation of insect-resistant transgenic crops expressing insecticidal proteins [33]. The major insecticidal protein used to develop genetically modified varieties is crystal protein (CRY) [34], also known as Bt protein because its gene is from *Bacillus thuringiensis* (Bt). Among these soybean cultivars, genetically modified cultivars expressing one or more Bt proteins have been widely deployed for pest management [34]. With the application of *Bt* gene in more crops, some studies have reported the emergence of pests resistant to Bt protein in maize, cotton, and others [35,36,37]. The resistance durability of the *Bt* gene has always been controversial. Plants have evolved endogenous insect resistance mechanisms during long-term interactions with pests. The adaptation of endogenous insect resistance gene can compensate for the deficiency of *Bt* gene [38]. Native insect resistance in soybean is a quantitative trait controlled by multiple genes of which the effect of a single gene on the trait is limited. Multiple soybean endogenous insect resistance genes have been reported [3,4,5,6,10,23]. To better apply these genes to improve insect resistance in soybean, the combined effects of genes on the trait needs to be evaluated. In this study, we identified a novel insect resistance gene, *GmCDPK17*, that acts differently from the known insect resistance gene, *GmCDPK38*. Both *GmCDPK17* and *GmCDPK38* were selected during the domestication of wild soybean to cultivated soybean. However, the percentages of resistance haplotypes for the two genes in cultivated soybean differ. The *GmCDPK38* resistance haplotype was widely used in cultivated soybean [23], while only a small number of cultivated soybean materials contained the resistance haplotype Hap1 of *GmCDPK17* (Figure 7d). Interestingly, cultivated soybeans carrying both resistance haplotypes had the highest insect resistance (Figure 8). Therefore, the introduction of the resistance haplotype of the *GmCDPK17* gene to soybean with the *GmCDPK38* resistance haplotype was expected to further improve insect resistance in cultivated soybeans.

### 3.3. GmCDPK17 May Have a Role in Other Biological Processes

*CDPK* genes are involved in multiple plant biological functions. Highly homologous genes tend to have the same functions. *GmCDPK17* is closely related to subfamily III orthologs *AtCPK10/30*, *OsCPK9* and *SlCDPK23* in Arabidopsis, rice and tomato, respectively [18,20,39,40]. In Arabidopsis, *AtCPK10* expression is induced by pathogen infection, and the T-DNA insertion line with *AtCPK10* expression results in a slight decrease in the levels of defense marker genes *PR1*, *PR2* and *AIG1* after inoculation with pathogens [28], illustrating that *AtCPK10* may be involved in the plant defense response. In addition to the defense response, *AtCPK10* plays an important role in abscisic acid- and Ca^2+^-mediated regulation of stomatal movements under drought stress through its interaction with heat shock protein 1 (HSP1) [41], whereas overexpression of *AtCPK30* strongly impairs root growth and endomembrane trafficking [42]. In addition, *AtCPK10* and *AtCPK30* phosphorylate the nitrate-responsive NIN-like protein transcription factor (NLP7) in the nucleus in response to nitrate [43]. In rice, *OsCPK9* is implicated as a positive regulator of drought tolerance and spikelet fertility [44]. Under drought stress, *OsCPK9* maintains water by increasing proline and soluble sugar contents and enhancing stomatal closure, while under normal conditions, *OsCPK9* improves spikelet fertility by regulating pollen viability. In tomato, the expression of *SlCDPK23* is induced by drought stress [40], suggesting its possible role in drought tolerance. All these results indicate that *GmCDPK17* orthologs from subgroup III broadly function as positive regulators of plant drought tolerance across plant species. In this study, we found that *GmCDPK17* has a similar function to Arabidopsis *CPK10* in regulating plant resistance to CCW. Overexpression of *GmCDPK17* in the Arabidopsis *cpk10* line rescued the phenotype of the mutant (Figure 3, Figure 4 and Figure 5). Moreover, further studies are needed to determine whether *GmCDPK17* has a role in other biological processes, particularly in regulating drought tolerance.

### 3.4. More Insect-Resistance Alleles May Exist in Accessions at Low Latitudes

Several studies have reported that the genetic diversity of genes is closely associated with their geographical distribution. The salt-tolerance haplotypes of the cation/H^+^ exchanger gene *GmSALT3* co-occurred with salt-affected soils [45]. The transposons in the CCT domain-containing genes *ZmCCT9* and *ZmCCT10* showed strong associations with latitude and were targeted by selection for maize adaptation to higher latitudes [46]. Li et al. [23] found that the resistance haplotype of *GmCDPK38* was mainly distributed at low latitudes in China, as determined by a geographic analysis. Likewise, in the present study, we found that the resistance haplotype of *GmCDPK17* in wild soybean and cultivated soybean subpopulations was also mainly distributed at low latitudes in China (Figure 7e). At these latitudes, soybeans are usually planted in intercropping systems, with a complicated farmland environment, rampant pests and changing conditions [47]. More generations of herbivorous pests are present at lower latitudes than at higher latitudes [48]. Therefore, plants growing at low latitudes face greater insect pest stress. As sessile organisms, plants must evolve sophisticated mechanisms to adapt to stressful environments, which may explain the existence of more endogenous insect resistance genes (haplotypes) in soybean at low latitudes. In addition, the coincidence of the insect-affected region and the insect-resistance haplotypes indicates that these alleles may be major selection factors determining the distribution and utilization of soybean, especially in regions with severe CCW attack.

## 4. Materials and Methods

### 4.1. Plant Materials and CCW Induction Treatments

The resistant accession PI and the susceptible accession QY were planted in a constant-temperature incubator with a photoperiod of 16 h/8 h at 26 °C. Soybean seedlings at the V4 stage (four nodes on the main stem beginning with the unifoliolate node) were used for CCW induction treatment as described in previous reports [49,50]. Total RNA was extracted from leaves of treated plants at 1, 6, 12, 24 and 48 h after CCW attack and control plants without CCW induction at the same time points. The resistant accession PI grown under conventionally managed natural field conditions was used for the tissue expression analysis. Roots, stems and leaves were sampled at the V4 stage, flowers were sampled at the R2 stage (one flower at a node immediately below the uppermost node with a completely unrolled leaf) [49], and seeds were sampled at 15 d after flowering to extract total RNA from each tissue.

Arabidopsis wild type (Col-0), mutant *cpk10* (SALK_032021C) from Arashare (https://www.arashare.cn/index/Product/index.html, accessed on 1 August 2021) and mutant *cpk28* (CS336535) from Arabidopsis Biological Resource Center (https://www.arabidopsis.org/index.jsp, accessed on 9 October 2013) were used as materials. *cpk10* and *cpk28* are mutants with a T-DNA insertion into the coding regions of *AtCPK10* (*AT1G18890*) and *AtCPK28* (*AT5G66210*), respectively. All Arabidopsis accessions were planted in a greenhouse at 24 °C and grown with a 14/10 h (day/night) photoperiod.

### 4.2. Prediction of Candidate Genes within Loci and Their Tissue Expression

Our group previously performed a genome-wide association analysis of 219 soybeans using 1142 SNPs and identified three adjacent SNPs on chromosome 6, BARC-025705-05000, BARC-025705-05001 and BARC-025707-05009, which were significantly associated with larval weight and larval duration [30]. The nearby genes within 130 kb upstream and downstream from these significant SNPs in the soybean reference genome (https://soybase.org/data/v2/Glycine/max/genomes/Wm82.gnm2.DTC4/, accessed on 15 June 2020) were selected as candidate genes, as described by Wang et al. [51]. By searching the expression data in SoyBase (https://soybase.org/, accessed on 22 June 2020), the expression patterns of candidate genes in distinct soybean tissues were predicted.

### 4.3. Cloning of GmCDPK17

The primers in this study were designed using Primer-BLAST (http://www.ncbi.nlm.nih.gov/tools/primer-blast/, accessed on 7 September 2020; Supplementary Table S5). *GmCDPK17* (*Glyma.06g189600*) was cloned from leaf complementary DNA (cDNA) of PI by PCR using specific primers. The PCR cycling conditions were as follows: predenaturation at 95 °C for 3 min; 35 cycles of denaturation at 95 °C for 15 s, annealing at 58 °C for 15 s, and extension at 72 °C for 1 min and 20 s; and a final elongation step at 72 °C for 5 min. The PCR product was purified and cloned into the T-vector using a pClone007 Blunt Simple Vector Kit (TsingKe, Beijing, China) and sequenced (Invitrogen, Shanghai, China). The recombinant T-vector was renamed T-GmCDPK17.

### 4.4. Gene Expression Analysis

Total RNA was extracted from Arabidopsis and soybean using the RNA Simple Total RNA Kit (Tiangen, Beijing, China), and first-strand cDNAs were reverse-transcribed with the PrimeScript^TM^ 1st Strand cDNA Synthesis Kit (TaKaRa, Dalian, China). The qRT-PCR was performed to analyze gene expression using an ABI 7500 system (Applied Biosystems, Carlsbad, CA, USA) with Aceq qPCR SYBR Green Master Mix (Vazyme Biotech Co., Nanjing, China). The relative expression levels of genes were analyzed using the 2^−ΔΔCt^ method [52]. Three biological and three technical replicates were used in these experiments. The soybean *GmTubulin* gene (*Glyma.03g124400*) and Arabidopsis *AtTubulin* gene (*At5g62690*) were used as internal controls to normalize the expression levels. The primers used for expression analyses are listed in Supplementary Table S5. Two-tailed *t*-test was used for statistical analyses.

### 4.5. Subcellular Localization

The *GmCDPK17* open reading frame (ORF) without a stop codon was cloned from T-GmCDPK17 and then ligated into the pFGC5941 vector containing the GFP gene downstream of the CaMV 35S promoter. The recombinant vector 35S:GmCDPK17-GFP and control vector 35S:GFP (empty vector) were transformed into *Agrobacterium tumefaciens* strain EHA105. The transformants were transformed into tobacco (*Nicotiana tabacum*) for transient expression by leaf infiltration [53]. The GFP signal in tobacco leaves was screened using confocal laser microscopy (Leica TCS SP2, Mannheim, Germany).

### 4.6. Constitutive Expression of GmCDPK17 and GmCDPK38 in Arabidopsis Mutants

The *GmCDPK38* gene (*Glyma.16G128600*) was cloned by Liu et al. [24]. The *GmCDPK17* and *GmCDPK38* sequences were cloned into the plant vectors pMDC83 and pBA002, respectively, under control of the CaMV35S promoter using specific primers (Supplementary Table S5). The recombinant plasmids were transformed into *Agrobacterium tumefaciens* strain EHA105, which was then used to transform Arabidopsis with the floral-dip method [54]. Through the above process, *GmCDPK17* and *GmCDPK38* were introduced into the Arabidopsis mutants *cpk10* and *cpk28* to generate T_1_ complementary lines (*35S:GmCDPK17/cpk10* and *35S:GmCDPK38/cpk28*, respectively), which was confirmed by PCR and qRT-PCR analyses (primers listed in Supplementary Table S5).

### 4.7. Overexpression and Suppression of GmCDPK17 in Soybean Hairy Roots

The pMDC83-based *GmCDPK17* overexpression (OE) vector (pMDC83-GmCDPK17) constructed above was used in this experiment. The Gateway method was used to construct the RNAi vector of the gene. A 190-bp fragment of the *GmCDPK17* coding region was cloned from T-GmCDPK17 using specific primers (Supplementary Table S5). According to the instructions of the Gateway^TM^ kit (Thermo Fisher Scientific, Shanghai, China), the fragment was connected to the pB7GWIWG2 vector to construct the recombinant RNAi vector pB7GWIWG2-GmCDPK17. Then, pMDC83-GmCDPK17, pB7GWIWG2-GmCDPK17 and their corresponding empty vectors (EV) (pMDC83 and pB7GWIWG2) were transformed into *Agrobacterium rhizogenes* strain K599. As described by Du et al. [55], the four transformed strains were separately inoculated into Jack cotyledons. The cotyledons were grown on White’s medium containing 500 μg mL^−1^ carbenicillin and 50 μg mL^−1^ cefotaxime at 25 °C in the dark to generate transgenic hairy roots. After 15 d, these transgenic hairy roots formed from the abdomen of inoculated cotyledons and were verified by PCR and qRT-PCR analyses (primers list in Supplementary Table S5). The transgenic hairy root lines from pMDC83-GmCDPK17, pB7GWIWG2-GmCDPK17, pMDC83, and pB7GWIWG2 were named OE-CDPK17, RNAi-CDPK17, OE-EV, and RNAi-EV, respectively.

### 4.8. Insect Force-Feeding and Free-Feeding Trials

Second-instar CCW larvae provided by the Jiangsu Academy of Agricultural Sciences (Nanjing, China) were starved for 12 h before trials. For the force-feeding trial of soybean hairy roots, equal amounts of fresh hairy roots of OE-CDPK17, RNAi-CDPK17, OE-EV and RNAi-EV were placed in plastic Petri dishes with wet filter papers to feed the CCW larvae for 6 d. Each genotype had 10 independent replicates, and each replicate contained five CCW larvae. The surviving CCW larvae were weighed at 0, 4, and 6 d. For the force-feeding trial of Arabidopsis, five CCW larvae were randomly placed on each 36-day-old plant of Col-0, *cpk10*, *35S:GmCDPK17/cpk10* T_1_, *cpk28*, and *35S:GmCDPK38/cpk28* T_1_ for 4 d. Each plant was separated and placed in a small netted room. Each genotype had five independent replicates. The surviving CCW larvae were weighed at 0, 2, and 4 d. The average larval weight was used to evaluate the resistance of each line (for each Arabidopsis line or soybean genotype). Two-tailed *t*-test was used for statistical analyses.

For the free-feeding trial on Arabidopsis, fully-expanded leaves of a similar size from 36-day-old plants of Col-0, *cpk10*, *35S:GmCDPK17/cpk10*, *cpk28*, and *35S:GmCDPK38/cpk28* were uniformly and randomly placed on the edge of a round Petri dish with wet filter paper. The Petri dish was 120 mm in diameter. Five 2nd instar CCW larvae were released in the center of the Petri dish to feed freely for 12 h. The leaf area was measured using a WinFOLIA^TM^ Pro LA2400 system scanner (Regent Instruments Inc., Sainte Foy, QC, Canada). The leaf area loss rate was used as an index to evaluate the CCW preference for different Arabidopsis lines. The trial was performed with five independent replicates.

### 4.9. GmCDPK17 Sequence Diversity Analysis

The whole-genome resequencing data from our population (121 wild soybeans, 197 landraces, 55 cultivars and 20 uncertain accessions) were obtained from studies by Lu et al. [56] and Li et al. [23] and used to analyze the polymorphic sites of *GmCDPK17* (including the 2000-bp promoter region, 188-bp 5′-untranslated region (UTR), 4047-bp exon and intron region, and 700-bp 3′-UTR) in cultivated and wild soybeans. A total of 34 heterozygous cultivated soybeans and sixty-nine heterozygous wild soybeans that had different bases detected at one site in the sequencing data were excluded from the haplotype analysis of our population. In addition, sequencing data from 302 soybean samples (62 wild soybeans, 130 landraces and 110 cultivars) downloaded from a database (https://figshare.com/articles/Soybean_resequencing_project/1176133, accessed on 17 November 2019) [57] were used to identify the *GmCDPK17* haplotypes among all except 76 heterozygous soybeans. The whole-genome sequencing data from our population (121 wild soybeans, 197 landraces and 55 cultivars) and the database population (62 wild soybeans, 130 landraces and 110 cultivars) were imported into the program VCFtools, which was used to calculate the nucleotide diversity (π), Tajima’s *D* and *F*_ST_ for the sequencing data within *GmCDPK17* (including the 2000-bp promoter region, 188-bp 5′-UTR, 4047-bp exon and intron region, and 700-bp 3′-UTR) between accessions in each subpopulation.

### 4.10. Phenotypic Data of the Population

The CCW larval weight data for 219 cultivated soybeans collected in Nanjing in 2009, 2013 and 2014 and 121 wild soybeans collected in Nanjing in 2014, 2016 and 2019 were derived from studies by Wang et al. [58], Liu et al. [30], Du et al. [59], and Li et al. [23]. In these insect bioassays, 2nd instar CCW larvae of a uniform size were randomly placed in a transparent plastic container and raised on the leaves from one soybean genotype. Insect weight data from the no-choice assays were collected after 7 days of feeding.

## 5. Conclusions

In this study, we cloned and identified *GmCDPK17*, a CDPK in subfamily III. Analyses of the expression pattern and transformation of Arabidopsis and soybean hairy roots indicated that this gene positively regulates plant resistance to common cutworm (CCW). Additional comparisons showed that the roles of *GmCDPK17* in insect resistance and plant growth and development were different from those of *GmCDPK38* in subfamily IV. Furthermore, the genetic diversity and evolution of *GmCDPK17* suggest that *GmCDPK17* might have been selected during soybean domestication. Soybean with resistance haplotypes of *GmCDPK17* and *GmCDPK38* exhibited high resistance to CCW. Overall, these results will help to improve the insect resistance of soybean.

## Data Availability

Not applicable.

## Supplementary Materials

The supporting information can be downloaded at: https://www.mdpi.com/article/10.3390/ijms232415696/s1.
