# Supplementary material for: Soybean CALCIUM-DEPENDENT PROTEIN KINASE17 Positively Regulates Plant Resistance to Common Cutworm (Spodoptera litura Fabricius)
Source: Int J Mol Sci. 2022 Dec 10;23(24):15696. doi: 10.3390/ijms232415696 (PMC9779107; doi:10.3390/ijms232415696)
Supplement: Supplementary file 1 [file ijms-23-15696-s001.zip › Supplementary Figures.pdf]

Supplementary Information

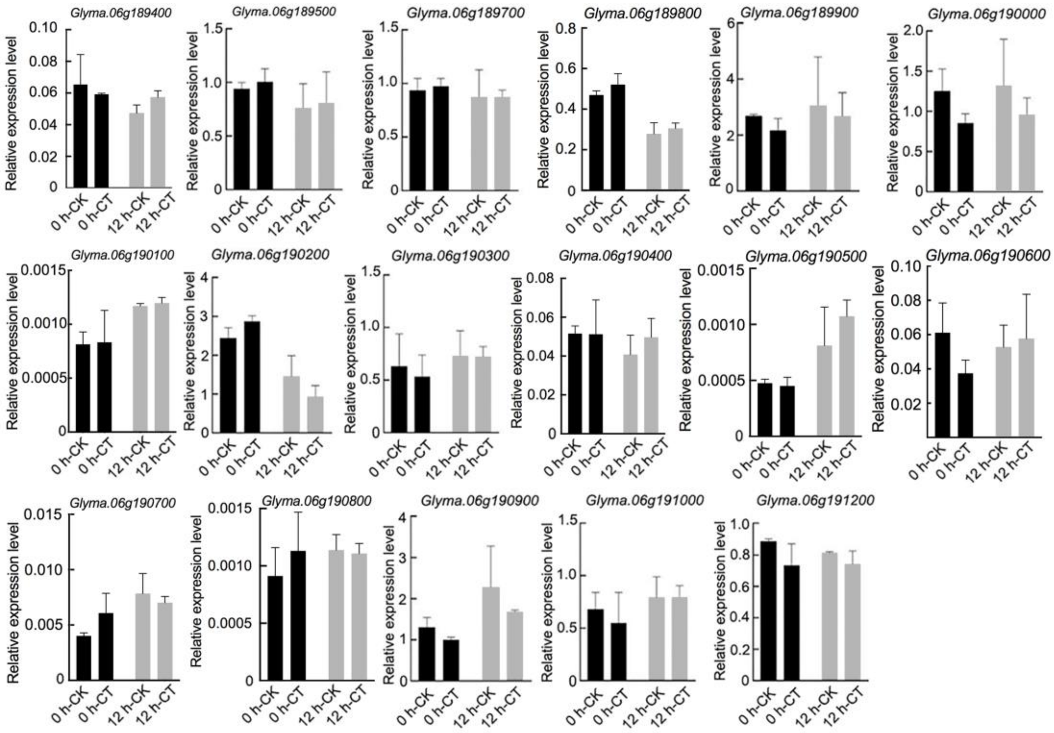

Figure S1. Transcriptional analysis of seventeen other genes in the QTL on chromosome 6 after CCW attack. 0 h-CK/CT, control and treatment before CCW induction. 12 h-CK/CT, control and treatment after CCW induction for 12 h (n = 3). Two-tailed t tests were used for statistical analyses. N = 3. Error bars denote ±SE.

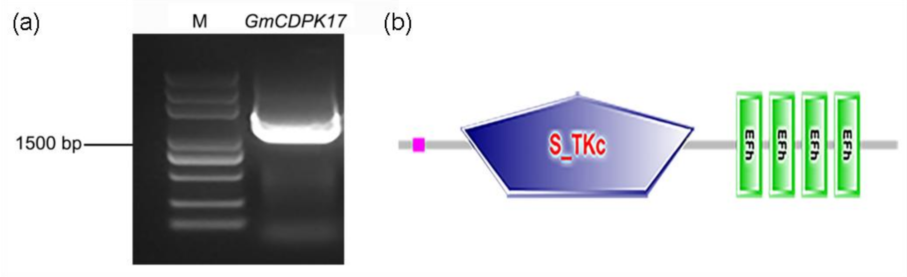

Figure S2. Cloning (**a**) and domain analysis (**b**) of the *GmCDPK17* gene. M: DNA Marker DL 2000. Purple box: N-terminal domain; S_TKc: serine/threonine protein kinase catalytic domain; EFh: EF chiral calcium-binding motifs.

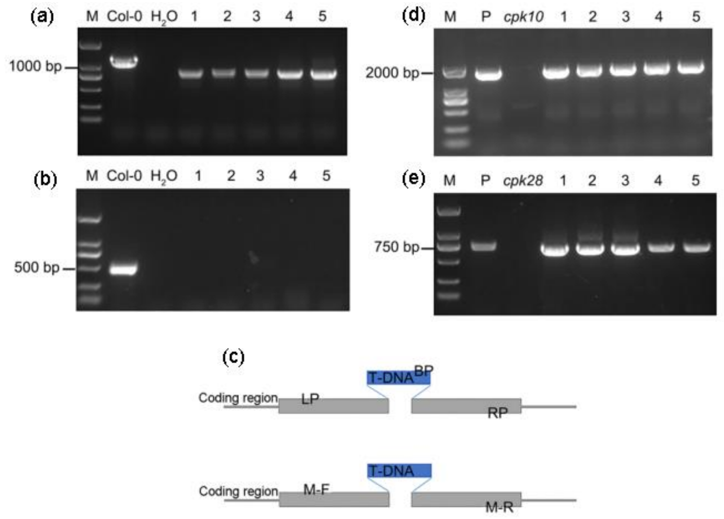

Figure S3. Identification of *Arabidopsis* plants using PCR. **(a)** Identification of *cpk10* mutants by PCR. Primers were designed according to three primer (LP, RP, and BP) detection methods near the T-DNA insertion site. 1-5: *cpk10* mutants. **(b)** Identification of *cpk28* mutants by PCR. Primers were designed near the T-DNA insertion site. 1-5: *cpk28* mutants. **(c)** T-DNA insertion diagram of the mutant. LP is the forward primer, and BP and RP are reverse primers for the identification of the mutant *cpk10* line (Supplementary Table 1). M-F and M-R: Forward and reverse primers for the identification of the mutant *cpk28* line (Supplementary Table 1). **(d)** Identification of *GmCDPK17* transgenic *cpk10* T_1 plants by amplifying a 1656-bp fragment of genomic DNA. **(e)** Identification of *GmCDPK38* transgenic *cpk28* T_1 plants by amplifying a 671-bp fragment of genomic DNA. M: DNA Marker DL 2000; Col-0: *Arabidopsis* ecotype Col-0; *cpk10*: *Arabidopsis cpk10* mutants; *cpk28*: *Arabidopsis cpk28* mutants; P: pMDC83-GmCDPK17 in **(d)** and pMDC83-GmCDPK38 plasmid DNA in **(e)** as positive controls; and H_2O as a blank control.

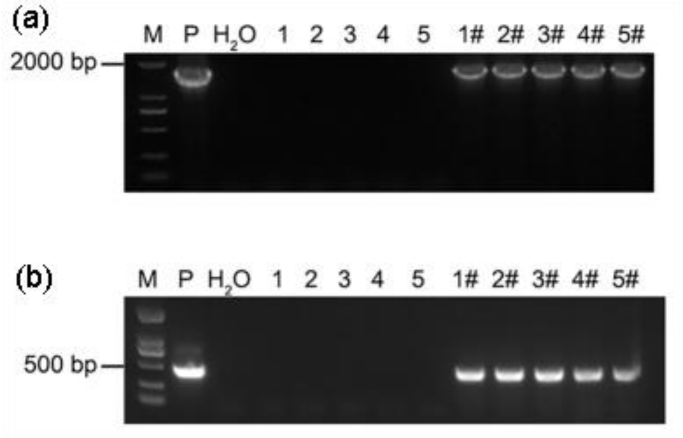

Figure S4. Identification of transgenic soybean hairy roots using PCR. **(a)** PCR amplification of *GmCDPK17* (1656 bp) in transgenic soybean hairy roots. P: pMDC83-GmCDPK17 plasmid DNA as a positive control; 1-5: soybean hairy roots transformed with the empty vector pMDC83 (OE-EV); 1#-5#: soybean hairy roots transformed with the pMDC83-GmCDPK17 plasmid (OE-CDPK17). **(b)** PCR amplification of the RNAi fragment of Gm*CDPK17* (378 bp) in transgenic hairy roots. P: pB7GWIWG2-GmCDPK17 plasmid DNA as a positive control; 1-5: soybean hairy roots transformed with the empty vector pB7GWIWG2 (RNAi-EV); 1#-5#: soybean hairy roots transformed with the pB7GWIWG2-GmCDPK17 plasmid (RNAi-CDPK17). M: DNA Marker DL 2000; H_2O as a blank control.
